# A 36-Year-Old Woman with Coronary Artery Dissection Two Weeks after Abortion 

**Published:** 2016-04-13

**Authors:** Arsalan Salari, Mahboobe Gholipur, Maedeh Rezaeidanesh, Anoosh Barzigar, Shahram Rahmani, Mohadeseh Pursadeghi, Hannan Ebrahimi

**Affiliations:** *Guilan Interventional Cardiovascular Research Center, Guilan University of Medical Sciences, Rasht, Iran.*

**Keywords:** *Coronary artery dissection, spontaneous*, *Abortion, spontaneous*, *Diagnostic techniques and procedures*

## Abstract

Spontaneous coronary artery dissection is a rare cause of acute coronary syndrome and sudden cardiac death. We report coronary artery dissection in a 36-year-old woman with retrosternal chest pain 2 weeks after abortion. Electrocardiography showed ST elevation in leads V2-V4 and ST depression in the inferior leads. Lab data were normal. Cardiac catheterization showed a suspicious thrombotic lesion at the proximal portion of the left anterior descending artery with a smooth contour consistent with distal haziness and dissection site. Final diagnosis was coronary artery dissection. At 1 week's follow-up, the patient was in good physical condition. At 1 month's follow-up, she had no complaints of discomfort. And finally, 8 months after having suffered a heart attack, she presented no evidence of angina, dyspnea, or congestive heart failure Spontaneous coronary artery dissection is a rare disease that mainly affects younger women. Compared with earlier reports, the prognosis seems to be improved by early diagnosis and interventional treatment.

## Introduction

Spontaneous coronary artery dissection (SCAD) may be the result of an intimal rupture with subsequent disruption of the vessel wall, leading to a double lumen (true and false lumens). Alternatively, bleeding of the vasa vasorum may result in an intramural hematoma. Progressive pressure-driven enlargement of the false lumen or intramural hematoma may cause further separation of the dissected layers, with the true lumen compression resulting in myocardial ischemia or infarction.^1^ SCAD is a rare cause of acute coronary syndrome and sudden cardiac death. The incidence of SCAD in the general population is between 0.28%^2^ and 1.1%,^3^ an estimation derived from the studies of consecutive patients with myocardial infarction undergoing coronary angiography. We report coronary artery dissection in a 36-year-old woman with retrosternal chest pain 2 weeks after abortion.

***Case presentation***

A 36-year-old woman (gravid 2, para 2, live birth 1) was admitted with retrosternal chest pain 2 weeks after having had an abortion. The patient had been well until the day of admission, when a sudden pain developing in her left arm and substernal areas awakened her from sleep at home. The chest pain was intense and very sharp and continued to exacerbate. Her symptoms resolved after a period of approximately 20 minutes. Thereafter, the pain recurred, associated with shortness of breath. She arrived at our hospital at 7 a.m. (5 hours after the initiation of pain). She had no known cardiovascular risk factors. The patient rated the pain at 7/10, and her blood pressure was 130/80 mm Hg. Electrocardiography showed a normal sinus rhythm with a rate of 86 beats per minute, ST-segment elevation (2 mm) in V_2_-V_4 _leads, hyperacute T wave in V_2_-V_4 _leads, and ST-segment depression in the inferior leads. Echocardiography was normal. Our main differential diagnoses were acute myocardial infarction, aortic dissection, and coronary artery dissection. Oxygen, morphine, Aspirin^®^, clopidogrel, enoxaparin, and sublingual nitroglycerin were administered. 

The patient underwent a full blood analysis, including blood count, coagulation profile, biochemistry, acute-phase reactants (ultrasensitive C-reactive protein, erythrocyte sedimentation rate, fibrinogen, rheumatoid factor, complement, and lipoprotein apolipoprotein A/B), lipid profile, thyroid function tests, and a full antibody screening (anti-nuclear, anti-DNA, anti-histone, anti-RNP, anti-SSB, anti-SSA, anti-Sm, anti-Scl 70, anti-Jo-1, anti-centromere, anticardiolipin, anti-myeloperoxidase, anti-protease, and anti-glomerular basement membrane antibodies). Immunoglobulins (IgG, IgA, and IgM) were also assessed. The lab data were normal. Cardiac catheterization through a femoral artery was performed at approximately 7:30 a.m. shortly after her admission.

**Figure1:**
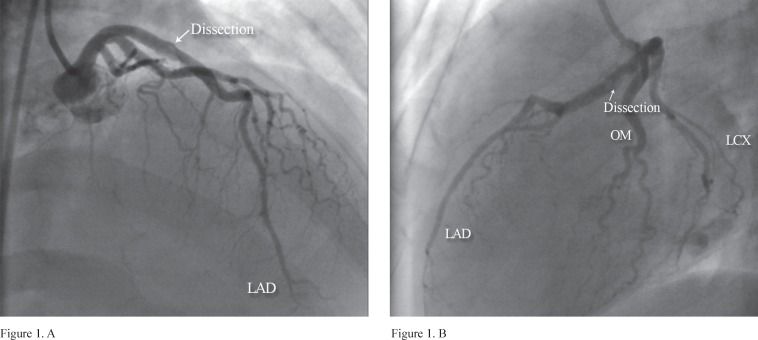
A (right anterior oblique cranial), B (left lateral view), and C (anteroposterior view) show a suspicious thrombotic lesion at the proximal portion of the left anterior descending artery with a smooth contour and a type C dissection with extraluminal extravasation of the contrast material.

LAD, Left anterior descending artery; LCX, Left circumflex artery; OM, Obtuse marginal

Multiple angiographic projections illustrated a suspicious thrombotic lesion at the proximal portion of the left anterior descending artery (LAD) with a smooth contour consistent with distal haziness and dissection site with the persisting extraluminal extravasation of the contrast material (dissection, type C), and minimal vascular disease in the other coronary arteries with the presence of coronary artery dissection (Fig. 1). The other coronary arteries were normal. The final diagnosis was coronary artery dissection. Percutaneous coronary intervention (PCI) was performed with a 3-15 XIENCE PRIME stent (Abbott Vascular) in the proximal portion of the LAD. She was discharged after 1 week with Aspirin^®^, metoprolol, clopidogrel, and atorvastatin. At 1 week's follow-up, she did not have any complaints; and at 1 month's follow-up, she was in good physical condition. And finally, 8 months after having suffered a heart attack, she presented no evidence of angina, dyspnea, or congestive heart failure.

**Figure 2:**
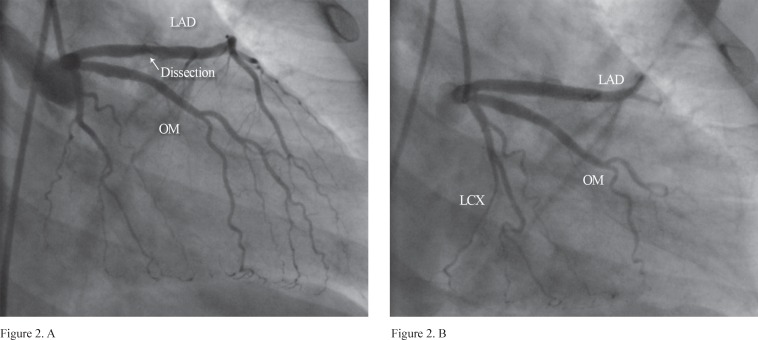
Angioplasty with stenting in the dissection site shows no residual dissection and extraluminal extravasation with a good result.

LAD, Left anterior descending artery; LCX, Left circumflex artery; OM, Obtuse marginal

## Discussion

The incidence of SCAD in the general population is between 0.28%^2^ and 1.1%,^3^ an estimation derived from studies of consecutive patients with myocardial infarction undergoing coronary angiography. Peripartum and postpartum SCADs are usually caused by multiple underlying factors, including sheer mechanical stress on the coronary artery during labor, prolonged coronary artery spasm, use of a uterine constrictor, and structural changes in the coronary artery medial wall that occur during pregnancy.^4^ It is established that pregnancy and the postpartum state are associated with several hormonal and hemodynamic changes that can persist for up to 6 months postpartum. Pregnancy is associated with an excess of progesterone, which results in the loss of the normal corrugation of elastic fibers, increase in the fragmentation of reticular fibers, and decrease in the amount of acid mucopolysaccharides, further reducing wall strength.^5^ These changes are normalized within 3 months of delivery.^6^ Treatment decision for SCAD is largely empirical, with the guidelines for the optimal management of SCAD having yet to be established. The published data describe different management strategies, including conservative treatment, PCI, coronary artery bypass grafting surgery,^7^ and heart transplantation. As the patients present with acute coronary syndrome, treatment with Aspirin^®^, Heparin, anti-ischemic, and fibrinolytic therapy is used as a part of the standard empirical pharmacological therapy for ST-segment elevation myocardial infarction.^4^^, ^^8^ However, fibrinolysis increases the flow into the false lumen and leads to the dissection.^9^ For coronary dissections, a critical concern is maintaining the patency of the true lumen. To that end, short-term use of a glycoprotein IIb/IIIa inhibitor has been reported as has long-term treatment with Aspirin^®^ and clopidogrel and Aspirin^®^ and enoxaparin. Be that as it may, although the patients in the previous case reports did well, whether the interventions were beneficial remains undetermined.^7^

PCI with stenting has become one of the choices of treatment in pregnant and postpartum patients^10^ with ongoing signs of ischemia^7^ and single-vessel disease, or in those in whom a large, viable myocardial territory is at risk such as proximal LAD disease. Because of the ongoing chest pain in our patient, we performed coronary intervention. The procedure was complication-free, and she was discharged after 7 days with Aspirin^®^, clopidogrel, metoprolol, and atorvastatin. At follow-up periods of 1 week, 1 month, and 8 months, she had no complaints of any discomfort. 

## Conclusion

SCAD is a rare disease that mainly affects younger women. Compared with earlier reports, the prognosis seems to have been improved by early diagnosis and interventional treatment. 

## Notes:

***This paper should be cited as: ***Salari A, Gholipur M, Rezaeidanesh M, Barzigar A, Rahmani S, Pursadeghi M, Ebrahimi H. A 36-Year-Old Woman with Coronary Artery Dissection Two Weeks after Abortion. J Teh Univ Heart Ctr 2016;11(2):98-101.
